# Reduced thrombin formation and excessive fibrinolysis are associated with bleeding complications in patients with dengue fever: a case–control study comparing dengue fever patients with and without bleeding manifestations

**DOI:** 10.1186/1471-2334-13-350

**Published:** 2013-07-28

**Authors:** Fernanda A Orsi, Rodrigo N Angerami, Bruna M Mazetto, Susan KP Quaino, Fernanda Santiago-Bassora, Vagner Castro, Erich V de Paula, Joyce M Annichino-Bizzacchi

**Affiliations:** 1 Hematology and Hemotherapy Center, University of Campinas, Rua Carlos Chagas, 480 – 13083-970, Campinas, SP, Brazil; 2 Epidemiological Surveillance Center, Hospital of Clinics, University of Campinas, Campinas, Brazil; 3 Department of Clinical Pathology, University of Campinas, Campinas, Brazil; 4 Department of Internal Medicine, University of Campinas, Campinas, Brazil

**Keywords:** Acquired coagulation disorders, Fibrinolytic disorders, Thrombin generation, Infectious diseases, Dengue fever, Dengue hemorrhagic fever, Thrombocytopenia

## Abstract

**Background:**

Dengue cases have been classified according to disease severity into dengue fever (DF) and dengue hemorrhagic fever (DHF). Although DF is considered a non-severe manifestation of dengue, it has been recently demonstrated that DF represents a heterogeneous group of patients with varied clinical complications and grades of severity. Particularly, bleeding complications, commonly associated to DHF, can be detected in half of the patients with DF. Although a frequent complication, the causes of bleedings in DF have not been fully addressed. Thus, the aim of this study was to perform a comprehensive evaluation of possible pathophysiological mechanisms that could contribute to the bleeding tendency observed in patients with DF.

**Methods:**

This is a case–control study that enrolled adults with DF without bleeding and adults with DF and bleeding complications during the defervescence period. Healthy controls were also included. Peripheral blood counts, inflammatory, fibrinolysis and endothelial cell activation markers, and thrombin generation were evaluated in patients and controls.

**Results:**

We included 33 adults with DF without complications, 26 adults with DF and bleeding and 67 healthy controls. Bleeding episodes were mild in 15 (57.6%) and moderate in 11 (42.4%) patients, 8 (30.7%) patients had bleedings in multiple sites. Patients with DF and bleedings had lower platelet counts than DF without bleeding (median = 19,500 vs. 203,500/mm3, P < 0,0001). Levels of TNF-α, thrombomodulin and VWF were significantly increased in the two dengue groups than in healthy controls, but similar between patients with and without bleedings. Plasma levels of tPA and D-dimer were significantly increased in patients with bleedings (median tPA levels were 4.5, 5.2, 11.7 ng/ml, P < 0.0001 and median D-dimer levels were 515.5, 1028 and 1927 ng/ml, P < 0.0001). The thrombin generation test showed that patients with bleeding complications had reduced thrombin formation (total thrombin generated were 3753.4 in controls, 3367.5 in non-bleeding and 2274.5nM in bleeding patients, P < 0.002).

**Conclusions:**

DF can manifest with spontaneous bleedings, which are associated with specific coagulation and fibrinolysis profiles that are not significantly present in DF without this complication. Particularly, thrombocytopenia, excessive fibrinolysis and reduced thrombin formation may contribute to the bleeding manifestations in DF.

## Background

Dengue is caused by a common arthropod-born virus with worldwide distribution. It is estimated that 50 million individuals are infected annually and 2.5 billion live in endemic areas [1]. Dengue is a febrile illness, with non-specific clinical manifestations that include fever, headache and myalgia, known as dengue fever (DF) [2]. Some patients, however, can manifest a severe form of the disease characterized by plasma leakage, thrombocytopenia, bleedings and shock, denominated dengue hemorrhagic fever (DHF) [3-7]. Although associated with DHF, bleeding complications may also occur in cases of DF [8]. In fact, it is estimated that about 50% of patients with DF can present bleeding episodes [9]. However, in contrast to patients with DHF, bleeding manifestations in patients with DF occur in the absence of plasma leakage [3,10]. Yet, the pathogenesis of bleeding complications in DF has not been fully addressed.

The aim of this study was to evaluate possible pathophysiological mechanisms that contribute to bleeding complications in adults with DF. We performed a comprehensive evaluation of hemostasis in a well-selected population of adults with DF, with and without bleeding manifestations. Particularly, the evaluation of blood coagulation included the thrombin generation test (TGT), a global hemostasis assay that mimics the physiological process of coagulation and is more specific to determine the integrity of clot formation [11]. Markers of fibrinolysis, inflammation and endothelial activation were also evaluated.

## Methods

### Study design and patients selection

This is a case–control study that included patients with suspected dengue infection with bleeding complications and patients without bleeding complications. Patients were selected during distinct outbreaks of dengue in the cities of Rio de Janeiro and Campinas, Brazil, in 2 different hospitals and 3 primary care medical centers. The study duration was from January 2008 until May 2011, but patients were included mainly in 2008 and 2010, when two important dengue outbreaks occurred in the Southeast Brazil, particularly in Rio de Janeiro and in Campinas, respectively. According to the Brazilian Ministry of Health the predominant circulating dengue serotype was DENV-2 in that period.

The inclusion criteria for the group with bleeding complications were suspected dengue infection, age over 17 years old, presence of spontaneous bleeding and being in the defervescence period. For the group without bleeding complications, the inclusion criteria were suspected dengue infection, age over 17 years old, no spontaneous bleeding and being in the defervescence period. The defervescence period was detected according to the medical follow-up at the primary care medical centers or at the hospitals; it was determined as the period when the body temperature tended to diminish. Usually, patients were enrolled for the study on the day they were tested for dengue serology (after the 5th day of fever), according to the Brazilian Ministry of Health protocol. Patients who met the inclusion criteria were reported to the study personnel who evaluated if the patients could be enrolled for the study. After the enrollment, patients were followed-up prospectively until recovery.

Exclusion criteria were the same for both groups: chronic kidney or liver disease, autoimmune or chronic infectious disease, hematological disorders and neoplasia. Pregnant women were excluded. Patients who lost follow-up after the enrollment were excluded. When the serology results were available, patients with negative anti-dengue IgM were excluded.

The serological tests for the detection of anti-dengue virus IgM and IgG antibodies were performed by enzyme-linked immunosorbent assay (ELISA) and were conducted at the referral laboratories of public heath. Patients with positive anti-IgM were diagnosed with dengue. All confirmed or discharged cases of dengue infection were in accordance with the disease notification by the official health authority.

The positive serological anti-dengue IgM test was used to confirm dengue diagnosis because the high sensibility of this test, above 80%, after the 5th day of symptoms. Also, the sensibilities of the virus antigen isolation or the virus acid nucleic detection by PCR are lower at the defervescence period, when our patients were enrolled for the study. In the Southeast Brazil other flaviviruses are not endemic and the population is not immunized against yellow fever virus, so the specificity of dengue serology is high.

**Figure 1:**
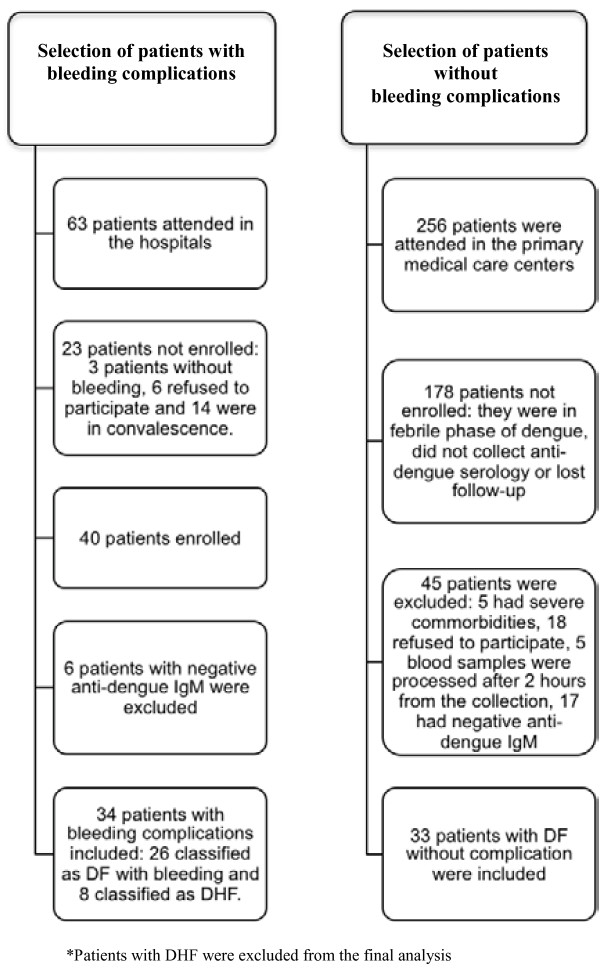
Flow chart of the study.

Three hundred and nineteen (319) adult patients were attended with suspected dengue infection in the period of the study, 63 patients were in the hospitals because of complications and 256 were attended in the primary care medical centers. From the 63 patients with complications attended in the hospitals, we were able to include in the study 40 patients because 3 had other complications than bleedings, 6 refused to participate and 14 had signs of convalescence (bleeding cessation and platelet arise). Anti-dengue IgM was positive in 34 out of 40 patients with bleeding complications included. From those, 8 were classified as dengue hemorrhagic fever, so 26 patients with dengue fever and spontaneous bleeding were included for the final analyses. From the 256 patients attended in the primary care centers, 178 patients were not enrolled for the study because they were in the febrile phase of dengue, did not collect anti-dengue serology or lost the follow-up. One patient had chronic myeloid leukemia, one patient had previous history of idiopathic thrombocytopenic purpura, three patients had chronic kidney disease, 18 refused to participate and five blood samples arrived in the laboratory after 2 hours from the blood collection, they were all excluded from the study. From the 50 patients included, 33 had positive anti-dengue IgM (Figure 1).

Healthy volunteers matched to patients by gender, age, ethnicity and ABO blood group were selected from the community as a control group. Exclusion criteria used for controls were equal to those used for patients.

The study was conducted in compliance with the Helsinki Declaration. This study was approved by the Research Ethics Committee of the School of Medical Sciences of the University of Campinas (UNICAMP) and was registered in the Brazilian National System of Information about Research Ethics (SISNEP) under the number CAAE = 0170.0.146.000-08 (http://portal2.saude.gov.br/sisnep/). A written informed consent was obtained from patients, or their attending relatives, and from controls.

### Patients’ clinical evaluation

Patients were prospectively evaluated by the same physician until they recovered from symptoms. Clinical evaluations and serial blood counts were perfomed from the enrollment day to the convalescence period to detect the changings in platelet counts and hematocrit. Previous blood counts from the febrile phase were retrospectively collected and used as basal parameters. The decreasing body temperature was evaluated clinically. Clinical and epidemiological data were recorded in a structured study form. Patients in the febril phase or in convalescence were not selected for the study.

Dengue cases were classified according to the World Health Organization (WHO) [7], by two different expert physicians, into DF or DHF. The following exams were performed for DHF diagnosis: serial hematocrit, chest X-ray, ultrasound and serum albumin levels. Blood counts were performed daily for inpatients and at intervals depending on the clinical course for outpatients. Chest X-ray, ultrasound and albumin were performed once during the hospitalization period. All patients with complicated dengue were hospitalized. Patients who were diagnosed with DHF on the day of the enrollment or during the follow-up were further excluded from the analysis, in accordance with our aim to evaluate only patients with DF (Figure 1).

Bleeding episodes were classified as mild, moderate and severe; this classification was adapted from WHO’s definitions [1]. Petechiae, ecchymosis and gingivorrhagia were considered mild bleeding symptoms. Bleeding episodes of gastrointestinal (GI) or genitourinary (GU) tracts were considered moderate. Bleeding episodes accompanied by hemodynamic instability were classified as severe bleedings.

### Laboratory procedures

Three tubes of blood samples were obtained from the participants at the time of their enrollment for the study. Two citrate and one EDTA-containing tubes were collected and samples were immediately centrifuged. Serum and platelet-poor plasma (PPP) obtained were separated and stored at −80°C within two hours from the collection. Control samples were processed following the same protocols and by the same investigator.

#### Markers of inflammation and endothelial cells activation

Serum levels of tumor necrosis factor (TNF)-α were performed to evaluate the degree of inflammatory activation. Plasma levels of thrombomodulin (TM) and von Willebrand factor (VWF) were evaluated as markers of endothelial activation. Commercial ELISA kits were used to determine serum levels of TNF-α (Quantikine HS, R&DSystems, Minneapolis, USA) and plasma levels of TM (Imunobind Trombomodulin ELISA kit, American Diagnostica INC, Stamford, USA). VWF antigen (VWF-Ag) levels in plasma were measured by ELISA as previously described [12].

#### Fibrinolysis pathways

Specific markers of fibrinolysis, such as tissue plasminogen activator (tPA), D-dimer and plasminogen activator inhibitor (PAI-1) were measured. Commercial ELISA assays were used to determine the plasma levels of D-dimer (IMUCLONE™ D-dimer, American Diagnostica INC, Stamford, USA), tPA (IMUBIND® tPA, American Diagnostica INC, Stamford, USA) and PAI-1 (PAI-1, American Diagnostica INC, Stamford, USA).

#### Thrombin generation test

Blood coagulation was evaluated by TGT in PPP using a commercial fluorescent assay (Technothrombin® TGA, Technoclone GmbH, Vienna, Austria) and fluorescence was read using FLx800 Fluorescence Microplate Reader (Bio-Tek Instruments, Winooski, USA). Four parameters of TGT were evaluated: the lag phase, which is the time from the beginning of the experiment to the first burst in thrombin formation, the thrombin peak, which is the maximum concentration of thrombin formed, the velocity-index, which is the amount of thrombin formed per minute and the total thrombin formed, represented by area under curve (AUC).

### Statistical analysis

Categorical variables were compared using Chi-square or Fisher exact test. To compare continuous variables between two groups Wilcoxon-Mann–Whitney test was used. To compare continuous variables among three or more groups Kruskal-Wallis analysis was used, followed by Tukey test to identify the differences, when necessary. The Spearman rank correlation was used to determine the correlation between variables. Multiple logistic regression and ROC curve were performed to identify variables associated to bleeding tendencies and to estimate optimal cut-off values, respectively.

Data were analyzed using SPSS for Windows version 10.0 (SPSS Inc, Chicago, IL, USA) and SAS System for Windows version 9.2 (SAS Institute Inc, Cary, NC, USA). Graphics were made using GraphPad Prism, version 4 for Windows (GraphPad Software Inc., La Jolla, CA, USA). P <0.05 was considered statistically significant.

## Results

Twenty-six adults with DF and spontaneous bleeding complications, 33 adults with DF without spontaneous bleeding and 67 healthy controls were included in the study. Eight patients had DHF and were excluded from the analysis. Twelve patients (20.3%) presented positive anti-dengue IgG serology.

Demographic parameters such as age, gender, ethnicity and ABO blood group were similar between patients and controls (Table 1). The most common commorbidity was hypertension, reported by 9 patients; patients with severe commorbidities were excluded.

**Table 1:** Demographic data of patients and controls

**Demographic data**	**Controls (n = 67)**	**DF without bleeding (n = 33)**	**DF with bleeding (n = 26)**	**P**
Female/Male (n)	35/32	13/20	15/11	0.32
Age (median and range), years	38.5 (19–76)	39 (17 – 71)	41 (20 – 79)	0.45
Caucasian/Afrodescendent (n)	38/29	24/9	12/14	0.11
ABO “non-O”/ ABO “O” (n)	36/31	19/14	10/16	0.30

This table shows the demographic parameters, as gender, age, ethnicity and ABO blood group evaluated in patients and controls.

Patients without bleeding complications were treated in the primary care medical centers where daily medical examination was performed and periodic blood counts obtained. In these centers, patients were treated with oral or parenteral hydration. Those with bleeding complications were hospitalized. Bleeding episodes were mild in 15 (57.6%) patients and moderate in 11 (42.4%) patients. Eight (30.7%) patients had bleedings in multiple sites. There were no severe bleedings. Patients with mild bleeding had predominantly disseminated ecchymosis and petechiae (n = 8), gingivorrhagia (n = 5) and epistaxis (n = 2). Patients with moderate bleeding had predominantly hypermenorrhea (n = 4), hematuria (n = 3), melena (n = 2), hemoptysis (n = 1) and hematemesis (n = 1). Only one patient received platelet transfusion. No other patient received blood transfusions. No hemostatic agents were administrated to the patients. Patients were mainly treated with crystalloids replacement. Table 2 contains the detailed clinical presentation of all patients.

**Table 2:** Clinical presentation of patients with dengue fever

**Clinical presentation**	**Patients with DF without bleeding (n = 33)**	**Patients with DF with bleeding (n = 26)**	**P**
Days with symptoms, median (range)	7 (5–10)	7 (2–15)	0.68
Days in the hospital, median (range)	0	2 (1–10)	N.A.
Fever	33 (100)	26 (100)	N.A.
Headache	17 (51.5)	22 (84,6)	0.01
Prostration	20 (60.6)	24 (92,3)	0.006
Nausea or Vomiting, n (%)	11 (33.3)	13 (50)	0.28
Abdominal pain, n (%)	3 (9)	12 (46)	0.002
Liver enlargement, n (%)	0	2 (7.7)	N.A.
Hypotension, n (%)	0	2 (7.7)	N.A.
Syncope, n (%)	0	2 (7.7)	N.A.
Acute renal insuficiency, n (%)	0	1 (3.8)	N.A.
Signs of plasma leakage, n (%)	0	0	N.A.
Shock, n (%)	0	0	N.A.

N.A. = not aplicable.

This table shows patients’ symptoms and signs of dengue at the first clinical evaluation or during the follow-up.

### Blood count parameters

**Figure 2:**
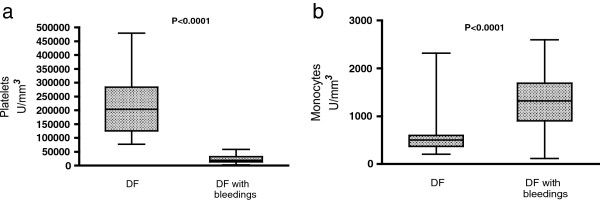
**Peripheral blood counts.** This figure illustrates the presence of thrombocytopenia **(a)** and monocytosis **(b)** in patients with bleeding complications. These parameters were performed in all patients. P values were calculated using the Wilcoxon-Mann–Whitney test. CT = controls.

Platelets and monocytes counts were significantly altered among patients with bleedings (Figure 2). Median platelet counts were 203,500/mm3 (range 77,000-479,000/mm3) in patients without bleedings and 19,500/mm3 (range 3,000-58,000/mm3) in patients with DF and bleedings (P < 0.0001). Median monocytes counts were 497/mm3 (range 203-2320/mm3) in patients without bleedings and 1,320/mm3 (range 496-2597/mm3) in patients with bleedings (P < 0.0001). The degree of thrombocytopenia was statistically correlated to the degree of monocytosis (r = −0.45, P < 0.0001).

Median hemoglobin levels were within the normal range among patient groups and there was no difference in hematocrit levels (median values = 43% vs. 43% in non-bleeding vs. bleeding patients, P = 0.48).

### Inflammatory and endothelial cell activation markers

**Figure 3:**
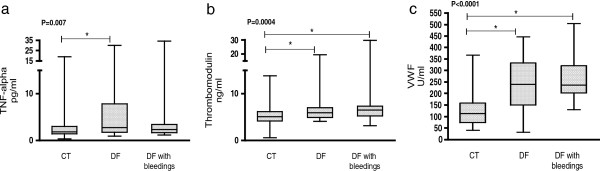
**Inflammatory and endothelial activation markers.** This figure illustrates the activation of the inflammatory response and the endothelial cells in dengue patients. Levels of inflammatory and endothelial cells activation markers were similar between DF groups. **a**. TNF-α, **b**. plasma TM levels. **c**. VWF antigen. These parameters were performed in all patients. P values were calculated using the Kruskal-Wallis analysis, followed by the Tukey post-test. CT = controls . * Place of differences detected by Tukey post-test.

Serum levels of TNF-α, plasma TM and VWF were increased in DF patients compared to controls. However, the levels of these markers were similar between non-bleeding and bleeding patients (Figure 3).

### Markers of fibrinolysis activity

**Figure 4:**
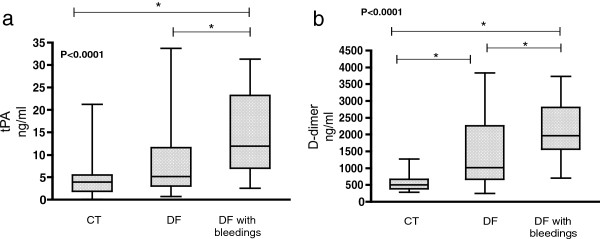
**Fibrinolysis parameters.** This figure illustrates the presence of hyperfibrinolysis in dengue patients. Fibrinolysis activity markers, such as tPA and D-dimer, were particularly elevated in patients with DF and bleeding complications. **a**. tPA levels; **b**. D-dimer levels. These parameters were performed in all patients. P values were calculated using the Kruskal-Wallis analysis, followed by the Tukey post-test. CT = controls. * Place of differences detected by Tukey post-test.

D-dimer and tPA levels were significantly increased in patients with bleedings. Median D-dimer levels were 515.5, 1028 and 1927 ng/ml in controls, non-bleeding and bleeding patients, respectively (P < 0.0001). Median tPA levels were 4.5, 5.2, 11.7 ng/ml in controls, non-bleeding and bleeding patients, respectively (P < 0.0001) (Figure 4). Plasma PAI-1 levels were similar among controls and DF groups (data not shown).

Thrombocytopenia was significantly correlated to higher D-dimer (r = −0.47, P < 0.0001) and tPA levels (r = −0.49, P < 0.0001) in patients.

### Blood coagulation parameters

Patients presented normal, or slightly altered, aPTT and PT. Median aPTT was 30.3” (range 27.5” – 47.5”) and median PT was 14.4” (range 13 – 24”). TGT was performed in 18 patients with DF (9 with bleedings and 9 without) and 24 controls. The first consecutive patients included in each group were selected for TGT analysis. All TGT parameters were significantly impaired in patients with bleedings, compared to controls. Particularly, decreased values of thrombin peak and AUC were observed in patients with bleedings (P = 0.014 and P = 0.008, respectively), suggesting that the thrombin formation is decreased in these patients (Table 3).

**Table 3:** Thrombin generation test

**TGT**	**Controls (n = 24)**	**DF without bleeding (n = 9)**	**DF with bleeding (n = 9)**	**P **^**#**^
Lag phase *	9.1 (1.8-17.7)	12.3 (3.1 -17.1) ^±^	11.6 (3.1 – 22.6) ^±^	0.021
Thrombin peak (nM) *	338.7 (145.1 - 512.2)	251.6 (70.7 – 432.3)	199.0 (15.9 – 394.1) ^±^	0.009
Velocity-index *	59.1 (13.3 – 128.7)	37.3 (6.7 – 193.2)	32.8 (12.6-73.0) ^±^	0.033
AUC *	3753.4 (2228.9–4905.6)	3367.5 (1512.9-4284.2)	2274.5 (180.5-3829.2) ^±^	0.002

* Median and range.

^#^ P values were calculated using the Kruskal-Wallis analysis.

^±^ Tukey test detected places of differences. In lag phase the differences were in DF with and also in DF without bleeding vs. controls. In thrombin peak, velocity index and AUC the difference was significant in the DF with bleeding group vs. controls.

This table shows the TGT results in controls and patients with DF, according to the presence of bleeding manifestations.

### Evaluation of independent parameters associated with bleeding tendencies

Using a multivariate logistic regression analysis, thrombocytopenia (OR = 200; 95%CI = 22-1000; P = 0.0003), monocytosis (OR = 13,5; 95%CI = 3,0-48,6; P < 0.0001) and elevated D-dimer (OR = 14,9; 95%CI = 2,4-90; P = 0.0032) were the strongest parameters associated to bleeding complications in DF.

ROC curve analysis showed that the cut-off values that better discriminated cases of DF with bleeding complications were platelets < 67,000/mm3 (P < 0.0001, 94% sensitivity and 93% specificity) and monocytes > 715/mm3 (P < 0.0001, 84% sensitivity and 82% specificity).

## Discussion

In this study, patients with dengue that did not fulfill the WHO criteria of DHF, and could otherwise be considered as having a non-severe form of the disease, indeed presented bleeding episodes and needed special medical care for that. This observation highlights that patients with DF can present bleeding complications and reinforces the importance of a detailed clinical evaluation of these patients in order to deliver appropriate care. Nowadays, efforts have been done to implement a new dengue classification, capable to identify signs of potential severe manifestations of the disease [1,2,8,10,13-16]. This new classification, however, is based only on clinical evaluations, many parameters are subjective and the varied mechanisms responsible for each detected complication are not fully understood. Understanding these mechanisms could promote the early identification of patients with severe disease and ultimately improve diagnostic and therapeutic strategies, thus limiting morbidity and mortality.

Bleeding complication is the most common complication reported in patients with DF. Particularly, adult patients may present more bleeding manifestations than children [17], and the bleeding manifestations in adults may occur in the absence of plasma leakage [18]. These observations raise at least two questions: first, that bleeding manifestations and plasma leakage may be two independent complications of dengue. Second, that the dengue hemorrhagic fever classification may underestimate some severe cases of dengue. In this study, we focused on evaluating the bleeding complication in adults with DF.

Thrombocytopenia has been considered as an important factor responsible for bleeding events in DHF [19] and as a common laboratory changing of DF [2,17]. The present study showed that thrombocytopenia was strongly associated with bleeding complications; raising the hypothesis that thrombocytopenia is not only a laboratory changing in DF but also an important cause of bleeding. Many factors can contribute to the onset of thrombocytopenia in DF, from a reactive immune response against the platelets to decreased platelet production [20-23], however the evaluation of the causes of thrombocytopenia is beyond the scope of our study. Besides thrombocytopenia, absolute monocytosis was also observed in patients with DF and bleedings.

Our results have demonstrated, as well, that the levels of TNF-α, TM and VWF were similar among DF patients, regardless of the presence of bleeding complications. These results were different from those previously described in DHF. Clinical studies have shown that markers of inflammation and endothelial cells activation may be increased in patients with DHF [5,24]. Indeed, the activation of the endothelial cells, by the virus or by inflammation, seems to be the initial pathogenesis of DHF [25]. Endothelial activation may be responsible for plasma leakage and shock [25-29]. However, our findings suggest that inflammation and endothelial cells activation may not be associated with bleeding complications of DF. Possibly, the fact that our patients with bleeding complications did not have an overt plasma leakage syndrome may explain the lack of increased inflammation and endothelial activation found in these patients, compared to those without bleedings.

On the other hand, excessive fibrinolysis was associated with bleeding complications in our patients with DF, since plasma levels of tPA and D-dimer were significantly increased in these patients. In a similar way, previous studies have demonstrated that patients with DHF may also present with excessive fibrinolysis [4,5,30-32]. It has been controversial, however, whether the stimulus for fibrinolysis in dengue was secondary to a disseminated intravascular coagulation (DIC) [4,17,33].

We failed to detect signs of DIC in patients with DF and bleedings. All blood smears from these patients were analyzed and none presented schistocytes. Results of aPTT and PT were within the normal range or only slightly increased. Furthermore, thrombin generation was reduced in patients with DF and bleeding.

Indeed, the finding of decreased thrombin generation is interesting since, to our knowledge, this coagulation abnormality has not been confirmed in dengue before. The relationship between dengue and activation of coagulation pathways is controversial [31]. Prolonged aPTT was demonstrated in some but not all clinical studies [4,9] while PT and thrombin time seem to remain unchanged [9]. We could confirm, by TGT, that the coagulation in patients with bleedings was impaired and the thrombin generation was decreased. However, the causes of this coagulation disorder remain speculative.

Clinical studies that evaluated the TGT in patients with chronic liver disorder demonstrated that, besides the prolonged aPTT and PT, the thrombin generation was normal [34]. The authors hypothesized that the decreased activity of the natural anticoagulants might compensate the impaired activity of coagulation factors in these patients, promoting thus a normal thrombin formation [35]. It is possible that a similar mechanism may happen in dengue and the activity of natural anticoagulants may be important in determining the thrombin formation. It has been shown previously that the activity of coagulation factors seem to remain within the normal range [33], although the activity of natural anticoagulants may be altered in some cases of dengue [5,30]. Plasma TM is a molecule that contributes to the activation of C protein [36], thus the increased levels of plasma TM observed in our patients with dengue could contribute to the activation this natural anticoagulant. Furthermore, it has been recently demonstrated that DV could bind directly to prothrombin and inhibit its activation to thrombin [37]. Regardless of the cause of the coagulopathy, our study showed that the coagulation activity may be decreased during dengue infections and that this disorder may be associated with bleeding complications.

Our results have demonstrated not only that cases of DF can manifest with spontaneous bleedings, but also that these cases may be accompanied by changes in peripheral blood counts, coagulation and fibrinolysis that may be not significantly detected in DF without this complication. In regard of bleeding complications, these results are consistent with the new concept that DF represents a clinically heterogeneous group of patients and also raise the hypothesis that different pathological mechanisms may be present in these patients. These findings corroborate the importance of the revision of dengue classification proposed by WHO in 2009.

It is important, also, to point out some limitations that may be found in this study. It is possible that some results have been influenced by the selection process and the sample size. This is a case–control study and patients were included as they presented or not the outcome of interest. However, a great number of patients could not be enrolled; this posed a problem to the selection process because some patients with possibly important clinical manifestations may have been missed. Of note, all patients with bleeding complications were selected from the hospitals and presented potentially severe manifestations of dengue, less severe cases were not detected among patients with bleedings. Because of that, it is possible that the severity of the coagulopathy associated with DF with bleedings may have been overestimated. This may also explain that, although being small, the sample size had enough power to show some differences in coagulation and fibrinolysis markers, particularly the profound differences in platelet counts observed between bleeding and non-bleeding patients. On the other hand, the careful selection approach warranted the standardization of the period of the disease analyzed and the well characterization of the groups. Another potential limitation is the fact that the dengue groups came from different medical settings. However the patients represent the same population, their follow – up and treatment were performed according to the WHO recommendations regardless of where they were treated. Although this study is not a controlled clinical trial, the methodology for patients’ diagnosis and classification used in this study reflects the medical care on dengue in the real world. Because of that, this study raises problems on dengue risk stratification that appear in the current clinical practices. Also, this study demonstrated some laboratory abnormalities that could be detected during the patients’ regular follow-up. The evaluation of patients in a real world set, without the need to follow a strict agenda of tests as in the controlled clinical trials, may represent an advantage because it allows the detection of laboratory changes that might possibly be reproduced in the clinical practice.

Moreover, our findings reflect dengue presentation and treatment in Brazil. The clinical presentation of dengue might be slightly different in Latin America than observed in Southeast Asia, because of the importance of non-dengue hemorrhagic fever syndromes for disease morbidity [8,16]. Therefore, our findings may raise important informations since the risk stratification of dengue has been reevaluated in order to englobe the disease presentation worldwide.

Finally, even with these limitations, our results highlighted that patients classified as dengue fever may present a complex coagulopathy and that the traditional dengue classification may not identify cases with laboratory abnormalities associated with potentially severe forms of the disease.

## Conclusions

Bleeding complications in patients with DF were associated with thrombocytopenia, reduced thrombin formation and increased fibrinolysis activity. The identification that some patients with DF may present with a complex coagulopathy reinforces the heterogeneity of this group and the necessity for new strategies to better stratify the severity of the disease for clinical and epidemiological purposes. Furthermore, understanding the bleeding disorders associated with dengue could lead to new diagnostic and therapeutic strategies in DF, since, until recently, bleeding manifestations had been studied mainly in patients with DHF.

## Abbreviations

Ng: Nanograms; Ml: Milliliters; Pg: Picograms; S: Seconds; mm3: Cubic millimeters; DV: Dengue virus; DF: Dengue fever; DHF: Dengue hemorrhagic fever; WHO: World Health Organization; aPTT: Activated partial thromboplastin time; PT: Prothrombin time; TGT: Thrombin generation test; tPA: Tissue plasminogen activator; PAI-1: Plasminogen activator inhibitor; TM: Thrombomodulin; VWF: Von Willebrand factor; TNF: Tumor necrosis factor; ELISA: Enzyme-linked immunosorbent assay.

## Competing interests

The authors declare that they have no competing interests.

## Authors’ contributions

FAO designed the study, enrolled patients, recorded clinical data, performed laboratory and statistical analysis, analyzed data and contributed to the manuscript production; RNA contributed with the design of the study, enrolled patients, recorded clinical data, contributed to data analysis and to manuscript production; BMM, SKP and FS performed laboratory analysis, VC contributed to patients enrollment and reviewed the manuscript, EVP contributed to the manuscript production, JMA designed the study, analyzed data and contributed to manuscript production. All authors read and approved the final manuscript.

## Pre-publication history

The pre-publication history for this paper can be accessed here:

http://www.biomedcentral.com/1471-2334/13/350/prepub
